# Occupational diseases in dentistry: Prevention

**DOI:** 10.4317/jced.64047

**Published:** 2026-06-29

**Authors:** Carmen López-Carriches, Ricardo Taheri, Victoria Mateos-Moreno, Alán García-Marqués, Cristina Meniz-García, Cristina Madrigal-Martínez-Pereda, Isabel Leco-Berrocal

**Affiliations:** 1 Associate Professor. Department of Dental Clinic Specialties. School of DentistryComplutense University of Madrid. Spain; 2 Doctor of Dental Surgery. DDS. Collaborator. School of Dentistry. Complutense University of Madrid. Spain; 3 Assistant Professor. Department of Dental Clinical Specialties. School of DentistryComplutense University of Madrid, Spain

## Abstract

**Background:**

Dentists, in the practice of their profession, are exposed to infectious diseases (most commonly hepatitis and HIV), biological accidents involving needles or sharp instruments and chemical intoxication from disinfectants, cements or dental resins. Also, they may suffer musculoskeletal disorders due to bad ergonomic postures, hearing loss and eye splashes or vision loss due to curing light, intense artificial lighting from dental units and constant visual strain in a limited space. Moreover, mental illnesses have been detected such as psychological issues like stress, fatigue, burnout, and depression. They are exposed to ionizing radiation and they may suffer from cancer more often than the general population.

**Materials and Methods:**

A bibliographic search was carried out in the following electronic databases: PubMed/MEDLINE and the Cochrane Library Plus.

**Results:**

Musculoskeletal problems, infectious diseases, visual and hearing impairments, and psychological disorders are some of the occupational diseases dentists may develop during their careers. A series of preventive measures are analyzed and proposed.

**Conclusions:**

The practice of dentistry carries the risk of various types of occupational diseases, which must be understood and prevented.

## Introduction

Dentists, during their professional practice, are exposed to a wide range of occupational hazards that can significantly impact their health and well-being. Among the most common risks are infectious diseases, particularly those transmitted through blood and saliva such as hepatitis B and C, and the human immunodeficiency virus (HIV). These infections often result from biological accidents involving needle sticks or injuries from sharp dental instruments (1 , 2). Additionally, chemical exposure is a frequent concern in dental clinics. Dentists are regularly in contact with potentially toxic substances such as disinfectants, dental cements and bonding resins. Repeated or prolonged exposure to these chemicals may lead to acute or chronic intoxication, allergic reactions, or respiratory and dermatological issues (1 , 2). Musculoskeletal disorders are also prevalent among dental professionals, largely due to prolonged static postures, repetitive movements, and inadequate ergonomic practices. These conditions frequently affect the neck, shoulders, back, and wrists, and can lead to chronic pain, reduced mobility, and even disability if not properly addressed through ergonomic intervention and preventive strategies (3). Dentists may also experience sensory impairments. Hearing loss is a known risk due to long-term exposure to high-frequency noise generated by rotary instruments, suction devices, and compressors commonly used in dental procedures. Likewise, the eyes are vulnerable to injury from chemical splashes, flying debris, or microbial contamination. Long hours of work in confined, brightly lit spaces, combined with the use of curing lights and other intense sources of artificial illumination, can contribute to visual fatigue and, in severe cases, vision impairment (4). Mental health disorders are another critical concern in the dental profession. High levels of occupational stress, demanding schedules, economic pressure, and the emotional burden of patient care contribute to conditions such as chronic fatigue, anxiety, burnout syndrome, and depression. These psychological issues not only affect the personal lives of dentists but also compromise the quality of care provided to patients (1 - 4). Finally, dentists are exposed to low doses of radiation. While individual doses are generally low, the cumulative effect over a career may increase the risk of developing certain types of cancer. The aim of this study is to inform practitioners about the various diseases that can significantly affect or shorten their careers, and to analyze whether their onset and progression can be prevented or delayed.

## Materials and Methods

A literature review was conducted using PubMed/MEDLINE and the Cochrane Library, including English-language articles on occupational diseases in dentistry with no publication year limits. The references cited in selected articles were also reviewed for additional sources.

## Results and Discussion

- Musculoskeletal Problems Dentists commonly suffer from musculoskeletal issues. The most affected areas include the neck, shoulders, lower back, upper back, and wrists (5 - 6). Dentistry involves high biomechanical risks due to forced postures and repetitive movements that damage joints. In a survey of Croatian dentists, 78.18% reported work-related upper back pain and 76.97% lower back pain. Carpal tunnel syndrome is also common (2). Particular attention should be paid to hand joint pain, as the hand is a dentist's main tool. A German study found that 20% of dentists had experienced hand pain in the past 12 months, requiring physiotherapy (7). In another study, 20% of female dentists reported thumb pain associated with radiographic evidence of osteoarthritis, suggesting a strong link to biomechanical factors (8). Ergonomics is essential for preventing and mitigating musculoskeletal problems in dental professionals. Without proper ergonomic practices, these physical demands can lead to chronic pain, reduced mobility, and even career-limiting injuries. To address this, dental clinics should prioritize physical ergonomics, ensuring that equipment, chairs, and instruments are properly designed and positioned to support healthy posture and reduce physical stress during procedures (9). Moreover, early education plays a vital role in prevention. Universities should incorporate comprehensive training in ergonomics, biomechanics, and postural awareness into dental programs (6). Teaching students how to maintain proper working positions and body mechanics can help prevent musculoskeletal disorders before they begin, supporting long-term health and career sustainability. - Infectious Accidents The risk of seroconversion varies depending on the virus: for hepatitis B virus (HBV), it ranges from 1-6% up to 22-31%, depending on the serological status of the source; for hepatitis C virus (HCV), the risk is approximately 1.8%; and for human immunodeficiency virus (HIV), it is about 0.3%(10 , 11). Among the most common and dangerous incidents are percutaneous injuries, particularly needle sticks, which pose the highest risk of transmission. Dentists are frequently injured while administering anesthesia or recapping needles, whereas hygienists are most often exposed during instrument cleaning (12). In a survey conducted among dentists in Mumbai, approximately 45% reported having experienced a needle stick injury (13). Aerosol-generating procedures further increase the risk of infection. These aerosols, created by rotary instruments and ultrasonic scalers, can contain blood, saliva, and microbial particles. If face masks become wet or are not properly fitted, their protective capacity diminishes significantly. FFP2 masks are recommended for superior filtration and protection (14). Given their close proximity to patients and repeated exposure to aerosols, dentists and hygienists are among the most vulnerable healthcare professionals to airborne and droplet-transmitted infections (15). Cross-infection from viruses like Herpes Simplex (HSV), Varicella-Zoster (VZV), Hepatitis B, C and D (HBV, HCV, HDV), Mycobacterium, Pseudomonas, Legionella, and resistant bacteria is also a serious concern in dental settings. Strict hygiene and vaccination protocols are critical in preventing cross-infection. There is limited research on the cross-transmission of HSV-1 in dentistry. All dental clinic staff should be vaccinated against hepatitis B to reduce the risk of transmission of this highly contagious virus, as patient-to-patient transmission is a possibility. The risk of hepatitis C transmission is lower. It has been shown that dental personnel have higher serum levels of antibodies specific to Legionella spp. compared to the general population, indicating occupational exposure to Legionella spp. through contaminated aerosols (16). All water reservoirs must be kept clean to prevent the growth of Legionella (17). All dental staff should operate under the assumption that every patient may be infectious. This universal precaution mandates rigorous infection control measures such as the use of sterilization indicators, spore testing, and validated disinfection protocols to ensure a safe clinical environment(18). During the COVID-19 pandemic, additional precautions were adopted. Patients were advised to rinse with chlorhexidine or diluted hydrogen peroxide before treatment. Rubber dams and antimicrobial agents were also used, though studies are inconclusive (19). - Chemical Exposure Risk In a dental clinic, various medications, disinfectants, and dental materials are routinely used, many of which carry the risk of chemical intoxication. These substances may not pose an immediate threat, but their effects are cumulative, and over time, prolonged or repeated exposure can result in toxic outcomes, potentially affecting the skin, respiratory system, or overall health (10). Among the most common allergens, latex gloves are a leading cause of skin reactions, particularly contact dermatitis. In addition, other substances such as detergents, disinfectants, lubricating oils, and certain dental materials can also provoke allergic responses in susceptible individuals (3). This highlights the need for proper ventilation, use of alternative hypoallergenic materials, and adherence to safety protocols to minimize occupational exposure. - Hearing Loss Hearing loss in dental professionals is primarily caused by excessive noise generated by high-speed rotary instruments, compressors, and suction devices (20). To detect early signs of hearing impairment, periodic audiometric testing is recommended (21). The use of hearing protection, such as earplugs, is advisable, and all rotary instruments should be properly maintained to minimize noise levels. Additionally, the use of sound-absorbing materials on clinic walls can help reduce ambient noise. Studies have shown that dentists have poorer hearing than control groups due to the constant exposure to noise from handpieces, compressors, and suction systems. According to research by Willershausen et al. (22), this occupational exposure significantly affects auditory function. Another study conducted by Al-Rawi (20) confirmed that dental assistants, laboratory technicians, and dentists all exhibit higher rates of hearing loss compared to non-exposed populations. The degree of hearing loss increases with longer daily exposure, typically more than 8 hours a day, and greater professional experience, especially in individuals with over 10 years in practice. In the long term, workplace noise contributes to reduced job performance and lower professional satisfaction. In the short term, acute noise levels in dental clinics can lead to headaches, fatigue, hypertension, irritability, and tinnitus (23 - 26). Although modern handpieces are quieter than older models, the overall noise environment in dental clinics remains a source of stress (27). Interestingly, the left ear is more commonly affected, as most dentists are right-handed, and thus the left ear is positioned closer to the suction device and handpiece. To address this issue, sound-absorbing materials should be incorporated into the design of dental clinics to help reduce noise levels and protect hearing (28). - Eye Damage The eyes can be damaged by mechanical, chemical, microbiological agents, or exposure to light. During the use of dental turbines, fluid splashes contaminated with microorganisms present in blood or saliva may reach the conjunctiva. Additionally, the eyes can be struck by tooth fragments, calculus, bone particles, metal pieces (such as steel or amalgam), or resin, all of which may travel at high speed. These impacts can result in anything from minor irritation to severe injuries, including blindness. Therefore, proper eye protection with appropriate safety glasses is essential, not only for the dentist but also for auxiliary staff and patients. The blue-light lamps used to cure composite materials can cause cellular damage to the retina. Protective glasses designed to block halogen or curing light should include side shields and be compatible with prescription eyewear (29). Another common source of ocular irritation is exposure to various chemical products used in dentistry, including adhesives, resins, disinfectants, eugenol, formaldehyde, eucalyptol, and alcohol. These substances can irritate not only the eyes but also the respiratory tract (29). In a survey conducted among 150 dentists, more than half reported experiencing some form of physical eye injury. Less frequent but notable conditions included conjunctivitis (27%), corneal abrasion (5%), retinal damage caused by chemicals, and cataracts (1.3%) (30). The use of lasers in dental procedures also poses a serious risk to vision. Laser equipment must always be used in combination with high-quality, specialized protective eyewear (3 , 10). - Psychological Problems Dentists face high levels of daily stress, often experiencing chronic fatigue and burnout syndrome. These conditions can lead to depression and anxiety and may contribute to the development of various psychosomatic illnesses, such as cardiovascular disease and hypertension, as mentioned earlier. Additionally, this mental and physical fatigue can impair a dentist's ability to concentrate and increase the risk of occupational injuries (10). Dentists often perceive their profession as highly stressful (3). This stress is frequently linked to patient interactions or economic pressures, which can lead to dissatisfaction with their profession (13). To maintain the ability to work effectively, it is essential to take care of one's health, including engaging in leisure activities. Proper management of work hours and workload, awareness of occupational risks, and prioritizing mental health are fundamental to sustaining a long and healthy professional life. - Risk of Miscarriage A slight association cannot be ruled out between exposure to various potentially toxic substances commonly found in dental clinics and an increased risk of miscarriage. These substances include acrylic compounds, mercury-containing amalgam, solvents, and disinfectants (31). - Cancer Risk The possible increased risk of cancer among dentists has not been thoroughly studied, despite the fact that ionizing radiation, known to cause leukemia at high doses, is regularly used in dental practice, along with exposure to a wide range of toxic substances. Interestingly, dentists tend to have lower rates of lung cancer, likely because they smoke less than the general population (32). However, radiologic technicians, nurses, radiologists, hygienists, and dentists are among the healthcare workers most frequently exposed to radiation, placing them at higher risk for certain types of cancer. Women in these professions are more prone to developing breast and thyroid cancer, while men are at greater risk for colon cancer (33). Although the radiation dose from intraoral X-rays is relatively low, each exposure carries a stochastic risk, meaning that even small doses can potentially lead to leukemia or other forms of cancer (34 , 35). To minimize radiation hazards, strict safety standards must be followed, standards that dentists are professionally obligated to know and apply. These include the use of personal dosimeters to monitor monthly radiation exposure, in accordance with established radiation protection protocols, although the detailed procedures are beyond the scope of this study (35).

## Conclusions

The practice of dentistry carries the risk of various types of occupational diseases, which must be understood and prevented. Emphasizing the importance of prevention, the study advocates for comprehensive protective strategies, including the use of personal protective equipment, proper ergonomic design, regular health monitoring, ongoing education in safety protocols, and attention to mental well-being, as essential measures to ensure a long, healthy, and sustainable career in dentistry.
